# Elevated CO_2_ and nitrogen addition have minimal influence on the rhizospheric effects of *Bothriochloa ischaemum*

**DOI:** 10.1038/s41598-017-06994-3

**Published:** 2017-07-26

**Authors:** Lie Xiao, Guobin Liu, Peng Li, Sha Xue

**Affiliations:** 1 State Key Laboratory Base of Eco-hydraulic Engineering in Arid Area, Xi’an University of Technology, Xi’an, 710048 China; 2 State Key Laboratory of Soil Erosion and Dryland Farming on the Loess Plateau, Institute of Soil and Water Conservation, Northwest A&F University, Yangling, 712100 China

## Abstract

The influence of elevated CO_2_ and nitrogen (N) addition on soil microbial communities and the rhizospheric effects of *Bothriochloa ischaemum* were investigated. A pot-cultivation experiment was conducted in climate-controlled chambers under two levels of CO_2_ (400 and 800 μmol mol^−1^) and three levels of N addition (0, 2.5, and 5 g N m^−2^ y^−1^). Soil samples (rhizospheric and bulk soil) were collected for the assessment of soil organic carbon (SOC), total N (TN), total phosphorus (TP), basal respiration (BR), and phospholipid fatty acids (PLFAs) 106 days after treatments were conducted. Elevated CO_2_ significantly increased total and fungal PLFAs in the rhizosphere when combined with N addition, and N addition significantly increased BR in the rhizosphere and total, bacterial, fungal, Gram-positive (G^+^), and Gram-negative (G^−^) PLFAs in both rhizospheric and bulk soil. BR and total, bacterial, G^+^, and G^+^/G^−^ PLFAs were significantly higher in rhizospheric than bulk soil, but neither elevated CO_2_ nor N addition affected the positive rhizospheric effects on bacterial, G^+^, or G^+^/G^−^ PLFAs. N addition had a greater effect on soil microbial communities than elevated CO_2_, and elevated CO_2_ and N addition had minor contributions to the changes in the magnitude of the rhizospheric effects in *B. ischaemum*.

## Introduction

Changes in global climate such as elevated CO_2_ concentrations, nitrogen (N) deposition, drought, and warming will have dramatic impacts on biological nutrient cycling in terrestrial ecosystems^1^. Soil microbial communities, especially rhizospheric microorganisms, serve as bridges between plants and soil, are key drivers of the global bio-geochemical cycles of mineral elements^2^. Much attention, however, has been given to characterize the responses of these communities to various scenarios of climate change^3–5^, but the responses of the communities to elevated CO_2_ and N deposition are poorly understood.

The individual effects of either elevated CO_2_ or N addition on soil microbial communities have been widely studied^6, 7^. CO_2_ concentrations are generally much higher in the pore spaces of soil (2000–3800 μmol mol^−1^) than in the atmosphere, so elevated CO_2_ usually influences the richness, composition, and structure of the communities indirectly, such as by increasing plant carbon (C) inputs to the soil and altering soil properties^7^. Phospholipid fatty acid (PLFA) analysis and denaturing gradient gel electrophoresis indicated that CO_2_ enrichment had no detectable effects on the composition and structure of the community^8–12^, but usually stimulated soil respiration^11, 13^. Analyses of functional genes indicated that elevated CO_2_ decreased the expression of genes involved in the C and N cycles^14, 15^, but Liu *et al*.^16^ reported that elevated CO_2_ increased the abundance of ammonia-oxidizing archaea and bacteria in a Chinese paddy field. These contradicting results might be due to the duration of CO_2_ enrichment^7^, the soil^15^, or the vegetation type^17, 18^.

N deposition can increase the concentrations of NH_4_^+^-N and NO_3_^-^-N in soil and improve the amount and quality of substrates available to soil microorganisms and thereby increase soil microbial biomass and activity^19^. Zhou *et al*.^20^ and Ma *et al*.^21^ reported that N additions increased the abundances of the microbial functional groups involved in the soil N cycle. N deposition, however, can also significantly decrease soil pH and thus dramatically decrease microbial biomass and the diversity of soil microbial communities^22–24^. The effects of N addition on these communities depended significantly on the amount added. Low or moderate N addition usually increases microbial biomass and diversity, but excessive N addition decreases them^25, 26^.

CO_2_ concentrations and N deposition have both increased under global climate change^27^. Elevated CO_2_ combined with N deposition increases litter decomposition, improves the supply of C to the soil ecosystem, and lessens soil N limitation, thereby substantially increasing C and N cycling in soil ecosystems^28, 29^. Soil respiration can also increase significantly under combined elevated CO_2_ and N addition^30^, more than for either alone^31^. Haase *et al*.^32^ found that neither elevated CO_2_ nor N supply affected the abundance of total and denitrifying bacteria in rhizospheric soil. Lee *et al*.^28^ recently reported that elevated CO_2_ and N addition affected bacterial and archaeal communities but not the fungal community. The effects of elevated CO_2_ and N addition on microbial communities vary with plant type^28^.

Processes in the rhizosphere mediate many important aspects of plant-soil interactions^33^. The activity and diversity of soil microbial organisms are generally higher in rhizosphere than bulk soil^34^. The release of low-molecular-weight organic compounds can change the biomass and composition of microbial communities, thus leading to further changes in rhizospheric microbial communities. Rhizodeposition is the main driver of rhizospheric effects^35^, defined as the ratios of the values of a variable in the rhizosphere and bulk soil; a positive (negative) rhizospheric effect indicates that a variable was higher (lower) in the rhizosphere than the bulk soil. Factors that regulate rhizospheric nutrient fluxes may control the magnitude of rhizospheric effects^4, 36^. A comprehensive understanding of the responses of soil microbial communities to elevated CO_2_ combined with N addition and exploring the changes of rhizospheric effects are important for understanding biogeochemical processes in terrestrial ecosystems in scenarios of global climate change.

*Bothriochloa ischaemum* (L.) Keng is a perennial herbaceous grass that is widespread in the hilly-gully regions of the Loess Plateau of China. It is characterized by quick reproduction and drought and trampling resistance and is a high-quality natural forage for livestock in this semiarid region. Its root system is well developed and forms a network that has a notable effect in preventing soil and water erosion. The soil of the plateau has low levels of total N^37^. The current rate of N deposition in this area is 2.2 g N m^−2^ y^−1^ ^38, 39^ and is expected to increase in the future^40^. Elevated global levels of CO_2_ and N deposition would synergistically lead to changes in the nutrient cycles in this temperate area. Elevated CO_2_ and N addition can significantly increase the photosynthesis of *B. ischaemum* and mitigate the N deficiency on the plateau^41, 42^. The response of soil microbial communities, especially rhizospheric microorganisms, to combined elevated CO_2_ and N addition, however, has rarely been studied. The purpose of the present study was to determine the response of soil respiration and microbial communities in the rhizosphere and bulk soil to an elevated CO_2_ level in combination with N addition. We hypothesized that (1) N addition would have a larger impact on community composition than elevated CO_2_ in the soil of this N-poor area, and (2) The effects of *B. ischaemum* rhizospheres on microbial-community variables should increase in the elevated-CO_2_ and N-addition treatments due to increased rhizodeposition.

## Results

### Soil TOC, TN, and TP contents

A two-way ANOVA showed that elevated CO_2_ did not have significant effect on SOC or TN content in either the rhizosphere or bulk soil, and N addition did not have significant effect on SOC, TN, or TP content (Table 1). Elevated CO_2_ and N addition did not have interactive effect on SOC, TN, or TP content. Elevated CO_2_ significantly affected TP content in both the rhizosphere and bulk soil (*P* < 0.05). TP content tended to decrease in response to elevated CO_2_ (Table 2).

**Table 1:** *P* values for the effects of elevated CO_2_ (C), N addition (N), and their interaction on chemical and microbial properties in the rhizosphere and bulk soil.

Indicator	Rhizosphere			Bulk soil		
	C	N	C × N	C	N	C × N
SOC content	0.270	0.820	0.879	0.235	0.221	0.700
TN content	0.267	0.970	0.865	0.453	0.906	0.712
TP content	**0.023**	0.085	0.565	**0.035**	0.200	0.659
BR	0.740	<**0.001**	0.204	0.441	0.340	0.769
SIR	0.561	0.785	0.504	0.131	0.533	0.920
AWCD	0.132	0.841	0.846	0.844	0.260	0.574
*H*	0.674	0.826	0.544	0.717	0.499	0.436
*D*	0.846	0.954	0.546	0.658	0.580	0.322
Total PLFA	**0.004**	**<0.001**	0.154	0.622	**<0.001**	0.932
Bacterial PLFA	0.087	**<0.001**	0.900	0.889	**<0.001**	0.878
Fungal PLFA	**0.007**	**0.004**	0.442	0.944	**0.020**	0.208
G^+^ PLFA	0.401	**0.010**	0.103	0.362	**0.002**	0.172
G^−^ PLFA	0.057	**0.001**	0.152	0.300	**<0.001**	0.097
Fungal/bacterial PLFA	0.074	0.364	0.522	0.925	0.424	0.140
G^+^/G^−^PLFA	0.240	0.404	0.837	0.170	0.139	0.439

Significant *P* values are highlighted in bold.

**Table 2:** Soil organic carbon (SOC), total nitrogen (TN), and total phosphorus (TP) contents (g kg^−1^) in the rhizosphere and bulk soil in the treatments.

Treatment	SOC content		TN content		TP content	
	Rhizosphere	Bulk soil	Rhizosphere	Bulk soil	Rhizosphere	Bulk soil
AN0	1.51 ± 0.13	1.48 ± 0.07	0.18 ± 0.02	0.19 ± 0.03	0.56 ± 0.01b	0.57 ± 0.01b
AN1	1.47 ± 0.14	1.53 ± 0.07	0.18 ± 0.02	0.19 ± 0.03	0.56 ± 0.02ab	0.57 ± 0.00b
AN2	1.44 ± 0.17	1.55 ± 0.12	0.19 ± 0.01	0.20 ± 0.02	0.54 ± 0.02ab	0.57 ± 0.01ab
EN0	1.54 ± 0.11	1.54 ± 0.02	0.20 ± 0.03	0.20 ± 0.03	0.55 ± 0.01ab	0.57 ± 0.01ab
EN1	1.53 ± 0.06	1.54 ± 0.06	0.19 ± 0.02	0.20 ± 0.02	0.53 ± 0.03a	0.56 ± 0.01ab
EN2	1.53 ± 0.19	1.58 ± 0.01	0.19 ± 0.02	0.20 ± 0.02	0.53 ± 0.03a	0.56 ± 0.01a

Different letters within a column indicate significant differences between treatments.

### Microbial respiration

**Figure 1:**
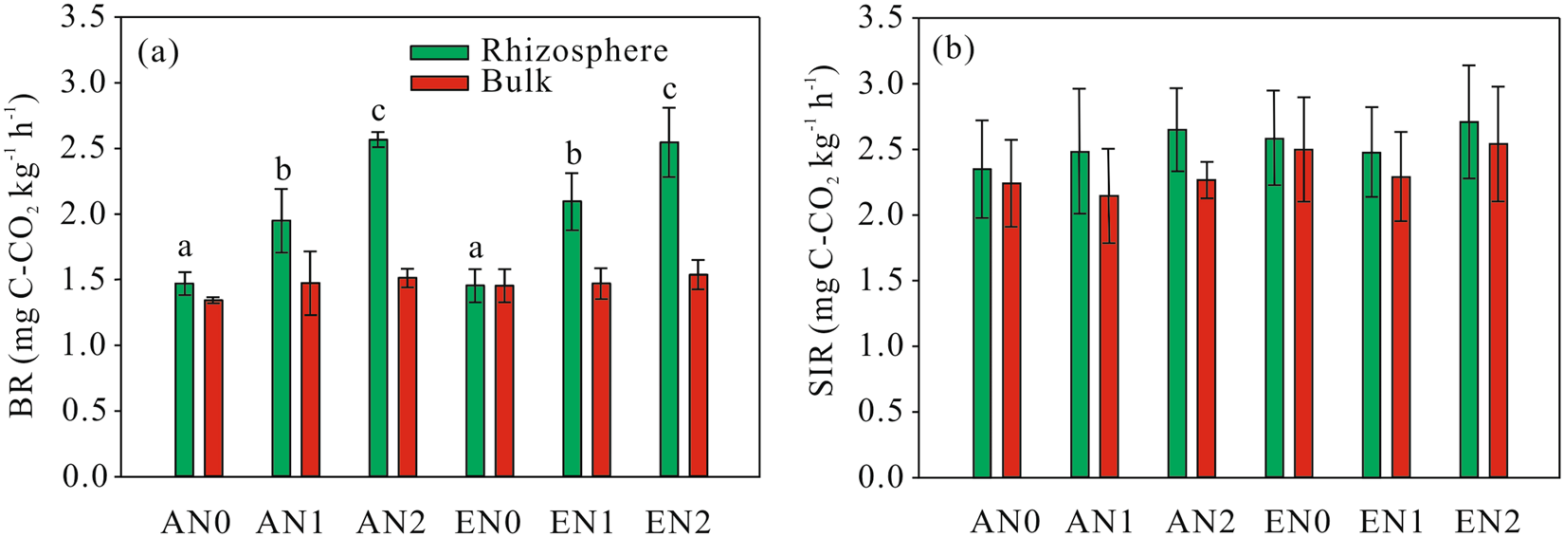
BR (**a**) and SIR (**b**) in the rhizosphere and bulk soil in the treatments. Different lowercase letters indicate significant differences between treatments in the rhizosphere. AN0, ambient CO_2_ and no N added; AN1 ambient CO_2_ and N supply at a rate of 2.5 g N m^−2^ y^−1^; AN2, ambient CO_2_ and N supply at a rate of 5 g N m^−2^ y^−1^; EN0, elevated CO_2_ and no N added; EN1, elevated CO_2_ and N supply at a rate of 2.5 g N m^−2^ y^−1^; EN2, elevated CO_2_ and N supply at a rate of 5 g N m^−2^ y^−1^.

**Figure 2:**
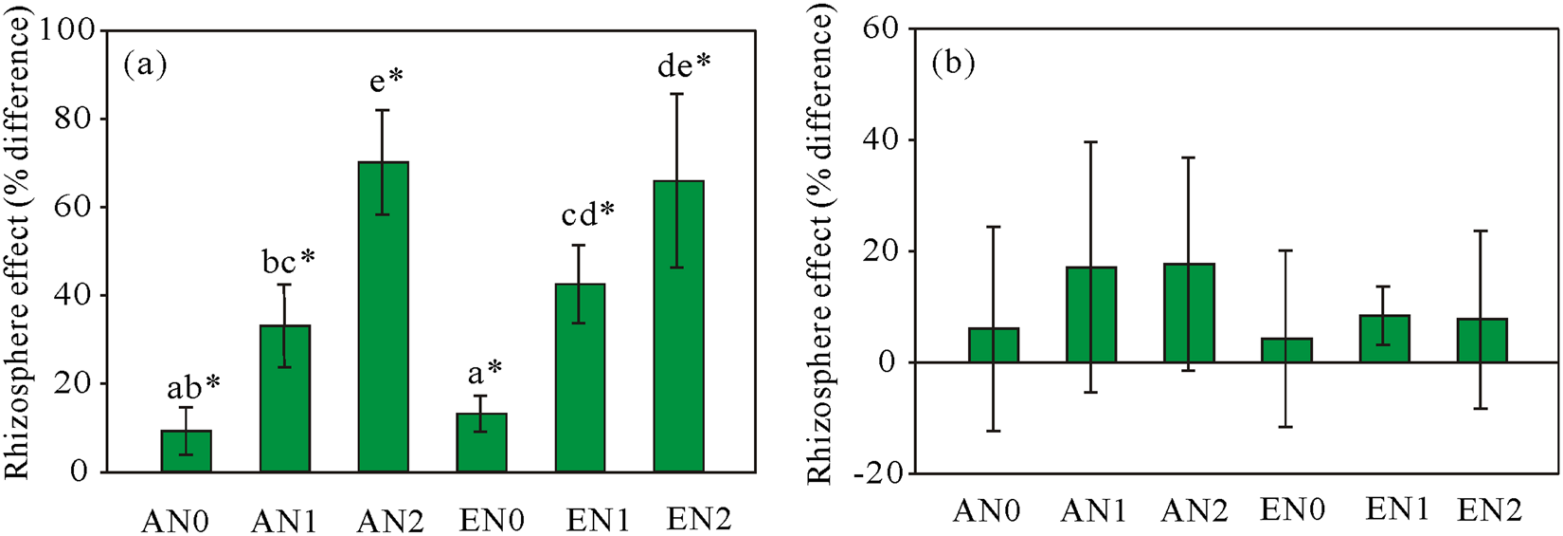
Rhizospheric effects for BR (**a**) and SIR (**b**) in the treatments. *Above each bar indicates significant difference between the rhizosphere and bulk soil. Different lowercase letters indicate significant differences in rhizospheric effects between treatments. AN0, ambient CO_2_ and no N added; AN1 ambient CO_2_ and N supply at a rate of 2.5 g N m^−2^ y^−1^; AN2, ambient CO_2_ and N supply at a rate of 5 g N m^−2^ y^−1^; EN0, elevated CO_2_ and no N added; EN1, elevated CO_2_ and N supply at a rate of 2.5 g N m^−2^ y^−1^; EN2, elevated CO_2_ and N supply at a rate of 5 g N m^−2^ y^−1^.

Elevated CO_2_ did not have significant effect on basal respiration (BR) or substrate-induced respiration (SIR) in the rhizosphere or bulk soil (Table 1). N addition significantly increased BR in the rhizosphere (*P* < 0.001). BR for the rhizosphere was 74.7 and 101.2% higher in N2 than N0 at ambient and elevated CO_2_, respectively (Fig. 1). BR was significantly higher in the rhizosphere than the bulk soil in all six treatments, and this positive rhizospheric effect was significantly affected by N addition (*P* < 0.001; Table 3, Fig. 2a). SIR was similar in the rhizosphere and bulk soil, indicating no rhizospheric effect (Figs 1b and 2b).

**Table 3:** *P* values for the effects of elevated CO_2_ (C), N addition (N), and their interaction on rhizospheric effects.

Indicator	C	N	C × N
BR	0.282	**<0.001**	0.175
SIR	0.339	0.601	0.875
Total PLFA	**0.010**	0.108	0.476
Bacterial PLFA	0.173	0.410	0.864
Fungal PLFA	0.057	0.365	0.685
G^+^ PLFA	0.921	0.633	0.337
G^−^ PLFA	0.051	0.186	0.120
Fungal/bacterial PLFA	0.140	0.557	0.497
G^+^/G^−^ PLFA	0.118	0.134	0.851

Significant *P* values are highlighted in bold.

### CLPP analysis

A two-way ANOVA indicated that neither elevated CO_2_ nor N addition had significant effect on AWCD, *H*, or *D* (Table 1). Elevated CO_2_ and N addition did not have interactive effect on the functional diversity of the soil microbial communities in either the rhizosphere or the bulk soil (Table 4).

**Table 4:** Average well color development (AWCD) and functional diversity (Shannon index (*H*), and Simpson index (*D*)) of the soil microbial communities in the treatments in the rhizosphere and bulk soil.

Treatment	AWCD		*H*		*D*	
	Rhizosphere	Bulk soil	Rhizosphere	Bulk soil	Rhizosphere	Bulk soil
AN0	0.24 ± 0.01	0.27 ± 0.02	2.76 ± 0.01	2.73 ± 0.02	0.93 ± 0.00	0.92 ± 0.00
AN1	0.23 ± 0.02	0.30 ± 0.02	2.74 ± 0.05	2.79 ± 0.03	0.93 ± 0.00	0.93 ± 0.00
AN2	0.24 ± 0.01	0.29 ± 0.01	2.79 ± 0.07	2.76 ± 0.05	0.93 ± 0.01	0.93 ± 0.00
EN0	0.26 ± 0.02	0.29 ± 0.01	2.75 ± 0.07	2.77 ± 0.04	0.93 ± 0.01	0.93 ± 0.00
EN1	0.25 ± 0.05	0.29 ± 0.02	2.81 ± 0.12	2.77 ± 0.08	0.93 ± 0.01	0.93 ± 0.01
EN2	0.27 ± 0.04	0.28 ± 0.02	2.78 ± 0.09	2.71 ± 0.10	0.93 ± 0.01	0.92 ± 0.01

### PLFA analysis

**Figure 3:**
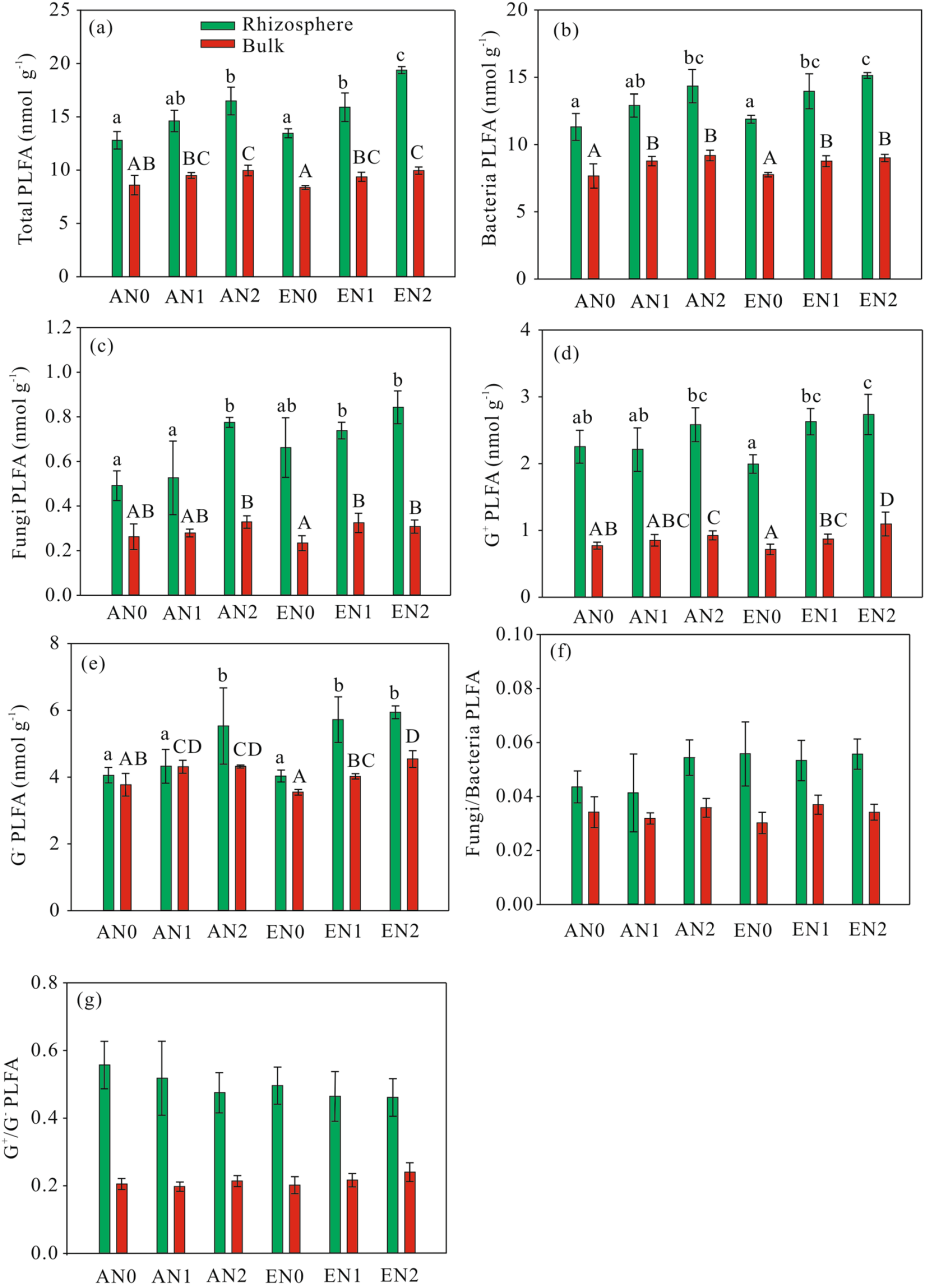
Total PLFA (**a**), bacterial PLFA (**b**), fungal PLFA (**c**), G^+^ PLFA (**d**), G^−^ PLFA (**e**), fungal/bacterial PLFA (**f**), and G^+^/G^−^ PLFA (**g**) in the rhizosphere and bulk soil in the treatments. Different lowercase letters indicate significant differences between treatments in the rhizosphere, and different uppercase letters indicate significant differences between treatments in the bulk soil. AN0, ambient CO_2_ and no N added; AN1 ambient CO_2_ and N supply at a rate of 2.5 g N m^−2^ y^−1^; AN2, ambient CO_2_ and N supply at a rate of 5 g N m^−2^ y^−1^; EN0, elevated CO_2_ and no N added; EN1, elevated CO_2_ and N supply at a rate of 2.5 g N m^−2^ y^−1^; EN2, elevated CO_2_ and N supply at a rate of 5 g N m^−2^ y^−1^.

**Figure 4:**
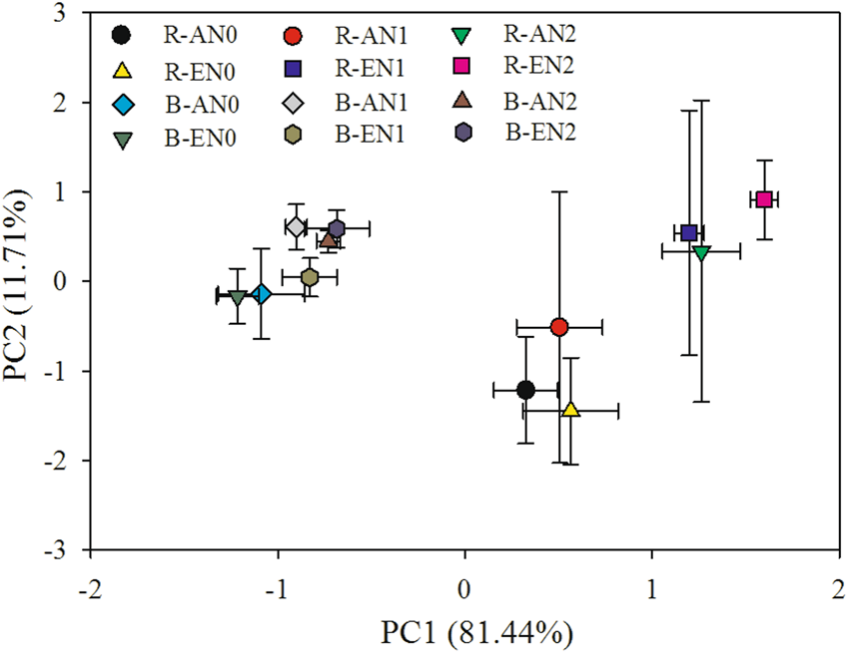
Principal component (PC) analysis of the PLFAs in the rhizosphere and bulk soil in the treatments. R, rhizospheric soil; B, bulk soil; AN0, ambient CO_2_ and no N added; AN1 ambient CO_2_ and N supply at a rate of 2.5 g N m^−2^ y^−1^; AN2, ambient CO_2_ and N supply at a rate of 5 g N m^−2^ y^−1^; EN0, elevated CO_2_ and no N added; EN1, elevated CO_2_ and N supply at a rate of 2.5 g N m^−2^ y^−1^; EN2, elevated CO_2_ and N supply at a rate of 5 g N m^−2^ y^−1^.

A two-way ANOVA indicated that elevated CO_2_ had significant effect on total and fungal PLFAs in the rhizosphere (Table 1). Compared with AN2, EN2 significantly increased total PLFA in the rhizospheric soil, and fungal PLFA was significantly higher in EN1 than AN1 in the rhizospheric soil (Fig. 3). N addition significantly increased total, bacterial, fungal, G^+^, and G^−^ PLFAs in both the rhizosphere and bulk soil (Table 1, Fig. 3). A principal component analysis of the PLFA data indicated that the first two components, PC1 and PC2, accounted for 81.44 and 1.71% of the variance, respectively (Fig. 4). The PLFA patterns differed significantly between the rhizosphere and bulk soil along PC1. Elevated CO_2_ and N addition did not have significant interactive effect on the composition of microbial PLFAs (Table 1).

**Figure 5:**
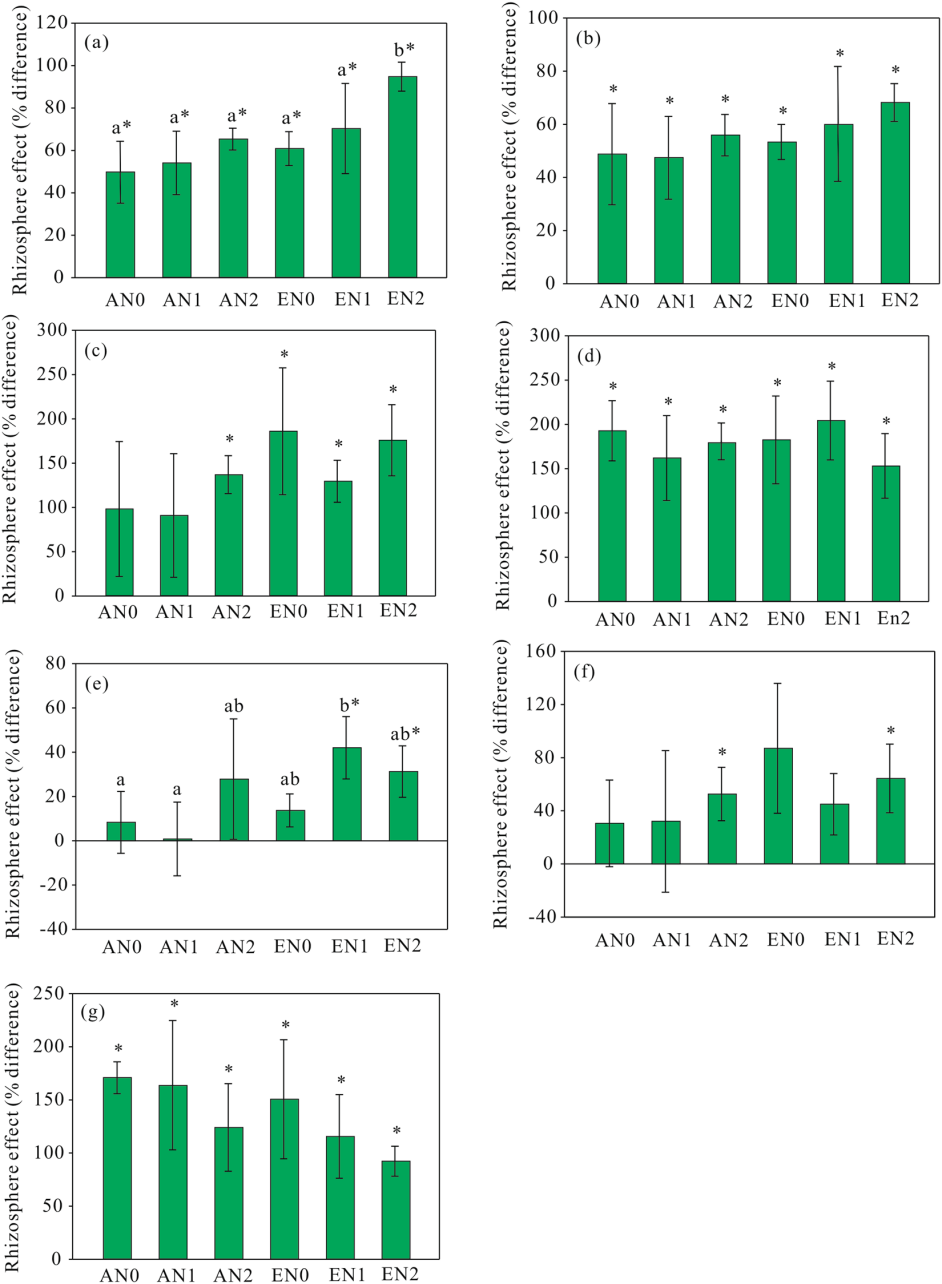
Rhizospheric effects for total PLFA (**a**), bacterial PLFA (**b**), fungal PLFA (**c**), G^+^ PLFA (**d**), G^−^ PLFA (**e**), fungal/bacterial PLFA (**f**), and G^+^/G^−^ PLFA (**g**) in the treatments. ^*^Above each bar indicates significant difference between the rhizosphere and bulk soil. Different lowercase letters indicate significant differences in rhizospheric effects between treatments. AN0, ambient CO_2_ and no N added; AN1 ambient CO_2_ and N supply at a rate of 2.5 g N m^−2^ y^−1^; AN2, ambient CO_2_ and N supply at a rate of 5 g N m^−2^ y^−1^; EN0, elevated CO_2_ and no N added; EN1, elevated CO_2_ and N supply at a rate of 2.5 g N m^−2^ y^−1^; EN2, elevated CO_2_ and N supply at a rate of 5 g N m^−2^ y^−1^.

Total, bacterial, G^+^, and G^+^/G^−^ PLFAs were significantly higher in the rhizosphere than bulk soil (Fig. 5). The positive rhizospheric effect (variables were higher in the rhizosphere than the bulk soil) for total PLFA was significantly increased only by elevated CO_2_ (Table 3). The positive rhizospheric effects for bacterial, G^+^, and G^+^/G^−^ PLFAs were not affected by either elevated CO_2_ or N addition.

## Discussion

### Characteristics of the soil microbial communities under elevated CO_2_ and N addition

Soil respiration is an important part of the global C cycle and the largest component of C flux from terrestrial ecosystems to the atmosphere^43, 44^. Elevated CO_2_ and N deposition can have profound impacts on soil respiration^30^. A meta-analysis found that N addition significantly increased soil respiration by 7.84% in grasslands^45^. N addition in our study significantly increased BR in the rhizosphere. This result was consistent with those by Luo *et al*.^46^ and Zhang *et al*.^47^, who found that N application significantly increased soil respiration, which they attributed to N-induced increases in plant growth, especially root biomass. We also found that N addition significantly increased root biomass (Table 5). Soil respiration is generally sensitive to elevated CO_2_^48^. Baronti *et al*.^13^ and Liu *et al*.^49^ observed increased soil respiration under elevated CO_2_. A meta-analysis by De Graaff *et al*.^50^ found that soil respiration increased by 17.1% under elevated CO_2_ and suggested that these increases could be due to microbial responses from changes in substrate availability. Elevated CO_2_ in our study did not have significant impact on BR in either the rhizosphere or bulk soil, suggesting that 106 days elevated CO_2_ might not significantly increase substrate availability to the community, although elevated CO_2_ significantly increased root biomass (Table 5).

**Table 5:** Effects of elevated CO_2_ concentration (C) and N addition (N) on plant biomass and nitrogen content.

Treatment	Total biomass (mg)	Shoot biomass (mg)	Root biomass (mg)	Root:shoot ratio	Nitrogen concentration (mg g^−1^)	Nitrogen content (mg pot^−1^)
AN0	466.67 ± 51.32a	280.87 ± 23.58a	185.8 ± 27.83a	0.66 ± 0.05a	4.75 ± 0.32a	2.21 ± 0.11a
AN1	1013.33 ± 247.86b	581.22 ± 153.55b	432.11 ± 97.7a	0.75 ± 0.08ab	5.37 ± 0.38b	5.46 ± 1.31b
AN2	2716.67 ± 358.38d	1473.87 ± 110d	1242.79 ± 249.93c	0.84 ± 0.11bc	5.31 ± 0.44ab	14.42 ± 1.83d
EN0	686.67 ± 75.06ab	406.55 ± 68.04ab	280.12 ± 18.14a	0.70 ± 0.10ab	4.77 ± 0.12a	3.28 ± 0.44a
EN1	1733.33 ± 94.52c	879.59 ± 54.56c	853.74 ± 41.76b	0.97 ± 0.03c	5.27 ± 0.34ab	9.12 ± 0.51c
EN2	4103.33 ± 290.23e	1966.89 ± 285.25e	2136.44 ± 178.17d	1.10 ± 0.19d	5.40 ± 0.26b	22.18 ± 1.73e
*P* values of two-way ANOVAs						
C	**<0.001**	**<0.001**	**<0.001**	**0.005**	0.969	**<0.001**
N	**<0.001**	**<0.001**	**<0.001**	**0.002**	**0.009**	**<0.001**
C × N	**<0.001**	0.130	**0.002**	0.201	0.841	**<0.001**

Different letters within a column indicate significant differences (*P* < 0.05) based on Duncan’s multiple range test. Significant *P* values are highlighted in bold. AN0, ambient CO_2_ and no N added; AN1 ambient CO_2_ and N supply at a rate of 2.5 g N m^−2^ y^−1^; AN2^,^ ambient CO_2_ and N supply at a rate of 5 g N m^−2^ y^−1^; EN0^,^ elevated CO_2_ and no N added; EN1, elevated CO_2_ and N supply at a rate of 2.5 g N m^−2^ y^−1^; EN2^,^ elevated CO_2_ and N supply at a rate of 5 g N m^−2^ y^−1^.

The response of the soil microbial community to elevated CO_2_ and N deposition depends on many factors, such as plant species, soil temperature and water content, and especially nutrient availability^23, 51^. Previous studies have reported that elevated CO_2_ or N addition increased, decreased, or had no significant impact on community structure^7, 15, 16, 26, 28, 52^. These contradictory results could be due to differences of microbial substrate availability under different scenarios of climate change. Elevated CO_2_ usually indirectly affects microbial communities by altering root biomass and exudation. Elevated CO_2_ for 106 days in our study significantly increased plant biomass, and elevated CO_2_ and N addition had significant interactive effect on plant root biomass, so total and fungal PLFAs significantly increased in the rhizosphere in the treatments with both elevated CO_2_ and N addition. N addition directly increased soil N content and may have indirectly improved nutrient availability by changing the conditions of plant growth (Table 5). The effects of N enrichment on the communities are associated with soil critical N loads or N saturation theories^53^. These theories propose that the effect of N enrichment on ecosystem functions would switch from stimulation to inhibition when the ecosystem reaches a critical N loading or saturation level^23, 54, 55^. The N-saturation theories suggest that low N additions usually increase soil microbial biomass and microbial diversity and that high N addition would decrease them^25, 26^. Plant growth on the Loess Plateau is restricted by soil N content, and N addition in our study significantly increased soil microbial biomass and shifted the community structure.

### Rhizospheric effects under elevated CO_2_ and N addition

Rhizospheres are zones of higher microbial turnover and activity, because they are adjacent to plant roots^56^. Many important aspects of plant-soil interactions are mediated by rhizospheric processes, including nutrient acquisition and root colonization by rhizospheric microorganisms. Microbial activity is higher and more diverse in rhizospheres than bulk soil^33^. We also found that BR and total, bacterial, G^+^, and G^+^/G^−^ PLFAs were significantly higher in the rhizosphere than bulk soil. The higher G^+^/G^−^ PLFAs in the rhizosphere than the bulk soil indicated that the rhizosphere communities were more heterotrophic via increases in C inputs when the plants were exposed to elevated CO_2_ and N addition^57, 58^.

Rhizospheric effects can be affected by elevated CO_2_ and N addition^36, 59^. The types and amounts of organic root exudates can be altered when plants are exposed to high levels of CO_2_, and these changes may affect rhizospheric microbial activity and community composition. Lee *et al*.^4^ reported that rhizospheric microbes responded to elevated CO_2_ more strongly than the microbes in bulk soil. Our results showed that elevated CO_2_ significantly increased the rhizospheric effects of total PLFA, as expected, because rhizospheric microbial communities are sensitive to elevated CO_2_. The rhizospheric effects of other microbial variables (such as bacterial PLFA, G^+^ PLFA, and G^−^ PLFA), however, responded weakly to elevated CO_2_. The impact of N addition on rhizospheric effects can be affected by many factors, such as plant species, soil type, soil chemical properties, and the amount and duration of N addition^35^. Phillips and Fahey^60^ found that N fertilization had a positive, negative, or no impact on rhizospheric effects for trees, depending on the tree species and soil variables. Ai *et al*.^61^ reported that long-term inorganic N addition reduced rhizospheric effects in a wheat-maize rotation system. N addition in our study did not significantly affect the rhizospheric effects for the soil variables, except BR, consistent with the results by Zhu *et al*.^36^, who also reported that N fertilization had minimal influence on the rhizospheric effects of two grass species. N is the limiting factor for plant growth on the Loess Plateau, and N addition in our study significantly increased plant total biomass and root biomass, so the root-derived C inputs to the soil (e.g. root exudates) may have been significantly affected by the N addition, and the available substrates in the rhizosphere (e.g. NH_4_^+^, NO_3_^−^) may also have been significantly absorbed by the plants (Table 5), which may account for the lack of significant shifts in the rhizospheric effects^34, 62^.

Evaluating the effects of elevated CO_2_ and N deposition on soil microorganisms in the rhizosphere and bulk soil is challenging because of their high diversity. Our PLFA analysis found that elevated CO_2_ and N addition had significant effects on the soil microbial community, especially in the rhizospheric soil. Both elevated CO_2_ and N addition contributed little to the changes in the magnitude of the rhizospheric effects, perhaps due to the low resolution of PLFAs for classifying soil microbial communities. As science and technology have developed, especially in the last decade, high-throughput molecular technologies have been developed for characterizing microbial communities, including high-throughput DNA/RNA sequencing, PhyloChio, GeoChip, mass spectrometry-based proteomics for community analysis, and metabolite analysis^63^. In future studies, these molecular methods maybe able to provide a more comprehensive understanding of microbial responses to scenarios of global climate change.

## Conclusions

N addition significantly increased BR in the rhizosphere and increased total, bacterial, fungal, G^+^, and G^−^ PLFAs in both the rhizosphere and bulk soil, but elevated CO_2_ only significantly increased total and fungal PLFAs in the rhizosphere when combined with N addition. These results demonstrated that N addition had a larger impact on the soil microbial communities than elevated CO_2_. Contrary to our second hypothesis, the rhizospheric effects of soil microbial variables were not significantly affected by elevated CO_2_ and N addition. These results suggest that the rhizospheres of *B. ischaemum* exert a more important control of community composition and structure than short-term elevated CO_2_ and N addition.

## Materials and Methods

### Plant species and soil used in the experiment

Seeds of *B. ischaemum* were collected in autumn 2013 from the natural grasslands at the Ansai Research Station (ARS) of the Institute of Soil and Water Conservation, Chinese Academy of Sciences (CAS), on the Loess Plateau of China (36°51′30″N, 109°19′23″E, 1068–1309 m a.s.l.). The rates of seed germination were >90% when germinated on moist filter paper in Petri dishes at 25 °C prior to the experiment.

A sandy-loam soil collected from the upper 20 cm of a farmland at ARS was used for this study. Soil gravimetric water content at field capacity (FC) and the wilting point were 20.0 and 4.0%, respectively. The soil organic C (SOC), total N (TN), and total phosphorus (TP) contents were 1.50, 0.21, and 0.57 g kg^−1^, respectively.

### Experimental design and sample collection

Each plastic pot (20 cm high, 15 cm in diameter) was separated vertically into two concentric zones, a central root zone and a root-free zone, by 25-μm nylon mesh bags (20 cm high, 9 cm in diameter) buried in the centers of the pots, enabling the passage of water and nutrients but not roots. The seeds of *B. ischaemum* were sown in the mesh bags on 1 June 2014. The soil-water content was maintained above 80% FC during the entire experiment.

The experiment began on 1 August 2014 after the seedlings were thinned to three per pot. The pots were transferred to two closed climate-controlled chambers (AGC-D001P, Qiushi Corp., Hangzhou, China) programmed at 13 h of light (28 °C, relative humidity (RH) of 50%, 300 μ (photons) m^−2^s^−1^) from 7:30 to 20:30 and 11 h of dark (22 °C, RH of 55%). The CO_2_ concentrations in the two chambers were maintained at 400 (ambient) and 800 (elevated) μmol mol^−1^ until the end of the experiment. An automatic control system was used to adjust the CO_2_ to the desired concentration in each chamber by regulating the influx rate of pure CO_2_ to the air blower. Each chamber housed three N-addition treatments (0 (control), 2.5, and 5 g N m^−2^ y^−1^). The N-addition treatments began on 18 August 2014. For each pot, NH_4_NO_3_ was dissolved in deionized water and then added to the pot soil, except for the control treatment where an equal volume of deionized water was added. N was added a total of six times during the experiment, with a frequency of every 15 days. The 0, 2.5, and 5 g N m^−2^ y^−1^ treatments received 0, 0.021, and 0.042 g NH_4_NO_3_, respectively, each time. All pots were weighed daily at 18:00, and water was added via plastic pipes to maintain soil-water contents above 80% FC. A total of six treatments were thus tested: ambient CO_2_ but no N added (AN0), ambient CO_2_ and 2.5 g N m^−2^ y^−1^ (AN1), ambient CO_2_ and 5 g N m^−2^ y^−1^ (AN2), elevated CO_2_ but no N added (EN0), elevated CO_2_ and 2.5 g N m^−2^ y^−1^ (EN1), and elevated CO_2_ and 5 g N m^−2^ y^−1^ (EN2). Each treatment had five replicates.

The experiment was completed on 15 November 2014. The soils of the root zone (adhering to the roots in the mesh bag) and the root-free zone (>1.5 cm outside the mesh bag) were collected and sieved through a 2-mm mesh. One subsample of each type of soil was air-dried, crushed, and passed through a 0.25-mm mesh for the determination of chemical properties, another subsample was stored at 4 °C for respiration and Biolog analysis, and a third subsample was stored at −20 °C for PLFA analysis.

### Soil chemical properties

SOC content was determined by wet digestion with a mixture of potassium dichromate and concentrated sulfuric acid, TN content was determined by the semimicro Kjeldahl method after digestion by H_2_SO_4_, and TP content was determined colorimetrically after wet digestion with H_2_SO_4_ + HClO_4_.

### Microbial respiration rate

The rate of soil microbial respiration was determined by CO_2_ emission^64, 65^. The basal respiration (BR) was determined by placing 10 g of each soil sample, moistened to 50–60% of the field capacity, in a hermetically sealed flask equipped with a rubber septum for gas sampling. The samples were then incubated at 28 °C for 7days under aerobic conditions, and the CO_2_ released was measured 0.5, 1, 2, 3, 4, 5, 6, 7 days after incubation with an infrared gas analyzer (QGS-08B, Befen-Ruili Analytical Instrument Co. Ltd., Beijing, China). Soil samples were amended with 0.6% (w/w) glucose before incubation for determining substrate-induced respiration (SIR). The BR and SIR data are expressed as μg CO_2_ g^−1^dw h^−1^.

### Community-level physiological profile (CLPP)

The CLPPs of the soil microbial communities were assessed using Biolog EcoPlates (Biolog, Hayward, USA) containing three replicates of 31 unique C substrates^66^. Briefly, soil samples were serially diluted to 10^−3^ suspensions in a sterile solution of 0.85% NaCl. The diluted suspensions were added to the Biolog EcoPlates, and all plates were incubated at 25 °C for one week. Color development was measured as optical density (OD) at 590 nm every 24 h using Microlog Rel 4.2 (Biolog, Hayward, USA). Negative optical densities or those under 0.06 were set to zero. The final OD of each well at 72 h was used to calculate the average well color development (AWCD), Shannon index (*H*), and Simpson index (*D*):

1 $${\rm{AWCD}}=\sum _{i=1}^{{\rm{n}}}\frac{({x}_{i}-c)}{31}$$

2 $$H=-\sum _{i=1}^{n}{p}_{i}(\mathrm{ln}\,{p}_{i})$$

3 $$D=1-\sum _{i=1}^{n}{({p}_{i})}^{2}$$

where *x*_*i*_ is the optical density measured at 590 nm for substrate in the EcoPlates, *c* is the OD of the control well, 31 is the number of C sources, *p*_*i*_ is the ratio of the absorbance in each well to the sum of absorbance for all wells, and *n* is the total number of C sources.

### PLFAs

The method for phospholipid extraction was adapted from Buyer *et al*.^67^. Briefly, 3 g of lyophilized soil were placed in a 30-ml centrifuge tube with a Teflon-lined screw cap. The fatty acids were directly extracted from the soil twice by adding 3.6 ml of citrate buffer (pH 4.0), 4 ml of chloroform, and 8 ml of methanol. The PLFAs were separated from neutral and glycolipid fatty acids by solid-phase-extraction chromatography. After mild alkaline methanolysis, the PLFA samples were qualitatively and quantitatively analyzed using an Agilent 7890 gas chromatograph (Agilent Technologies, Santa Clara, USA) equipped with an autosampler, split-splitless injector, and flame ionization detector. The system was controlled with Agilent ChemiStation and MIDI Sherlock software (Microbial ID, Inc., Newark, USA). An external standard of 19:0 methyl ester was used for quantification.

We selected the following PLFA signatures to serve as indicators of specific microbial groups: iso- and anteiso-branched fatty acids for Gram-positive (G^+^) bacteria^68^, monounsaturated and cyclopropyl 17:0 and 19:0 fatty acids for Gram-negative (G^−^) bacteria^69^, and 18:2w6c for fungi^70^. Total biomass was obtained by summing the concentrations of all fatty acids detected in each soil sample.

### Statistical analysis

All results are expressed as means ± standard deviations. Rhizospheric effects were calculated as the percent difference between the rhizospheric and bulk-soil samples for each measured variable^34, 36^. Student’s *t*-tests were used to compare the values between the rhizosphere and bulk soil to indicate the statistical significance of the calculated rhizospheric effect. SOC, TN, and TP contents and microbial functional diversity did not differ significantly between the rhizosphere and bulk soil, so we have not reported the rhizospheric effects for these variables. Two-way analyses of variance (ANOVAs) at a probability level of 0.05 were used to assess the effects of elevated CO_2_, N addition, and their interaction on the biochemical properties of the rhizospheric and bulk soil and the rhizospheric effect for each variable. Means were compared using Duncan’s multiple range test for significant differences (*P* < 0.05). A principal component analysis examined the PLFA community structure among the treatments. The above statistical analyses were performed using SPSS 16.0 (SPSS Inc., Chicago, USA).
